# Automated Stuttering Detection Using Deep Learning Techniques

**DOI:** 10.3390/jcm14103552

**Published:** 2025-05-19

**Authors:** Noura Alhakbani, Raghad Alnashwan, Abeer Al-Nafjan, Abdulaziz Almudhi

**Affiliations:** 1 Information Technology Department, College of Computer and Information Sciences, King Saud University, Riyadh 11543, Saudi Arabia; nhakbani@ksu.edu.sa (N.A.); 444203361@student.ksu.edu.sa (R.A.); 2 Computer Science Department, College of Computer and Information Sciences, Imam Mohammad Ibn Saud Islamic University, Riyadh 11432, Saudi Arabia; 3 Department of Medical Rehabilitation Sciences, College of Applied Medical Sciences, King Khalid University, Abha 62529, Saudi Arabia; almudhi@kku.edu.sa

**Keywords:** stuttering detection, deep learning, speech pathology, machine learning

## Abstract

**Background/Objectives:** Disfluencies such as repetitions, prolongations, interjections, and blocks in sounds, syllables, or words can sometimes hinder communication. Currently, disfluencies are manually measured, which has inherent limitations, such as being time-consuming and subjective, which can lead to inconsistencies in measurement. **Methods:** To address these challenges, this study presents an innovative automated system for detecting disfluencies utilizing advanced artificial intelligence technologies; specifically, deep learning models such as convolutional neural networks (CNN) and convolutional long short-term memory (ConvLSTM). The system was evaluated using two benchmark datasets: FluencyBank and SEP-28K. **Results:** Our proposed system demonstrates remarkable performance, achieving detection accuracies of 0.97 and 0.96, respectively, for CNNs and ConvLSTM models. These results not only exceed those of prior studies but also highlight the effectiveness of our approach in enhancing stuttering evaluation. **Conclusions**: By providing a reliable and efficient tool for professionals in therapeutic settings, our system represents a significant advancement in the field, offering improved outcomes for individuals affected by stuttering.

## 1. Introduction

Speech is a fundamental medium for conveying ideas and emotions. Not all individuals possess the capacity for flawless, fluent communication [1]. The efficacy of speech lies in its fluency, which denotes the natural flow between the phonemes, syllables, and words that constitute a message [2,3]. The most common disfluencies observed in speech are repetitions, prolongations, interjections, and blocks (abnormal stoppages) observed at the phoneme, syllable, or word levels [1,4]. These disfluencies hinder the smooth flow of speech and pose a notable challenge for >80 million people globally, equivalent to approximately 1% of the world’s population [2].

These disfluencies have been categorized as normal disfluencies and disfluencies observed in the disordered population. The type and number of disfluencies observed in normal disfluencies and disfluencies observed in the disordered population are different [1]. Compared to normal disfluencies, disordered disfluencies can substantially disrupt the flow of speech at a considerably higher rate than typical disfluency [5]. One of the major challenges is identifying disfluencies using subjective approaches. Conventionally, disfluencies are manually counted and divided by the total number of spoken words. However, subjective assessments are prone to inconsistencies, can be lengthy, and are susceptible to errors [6]. Another method involves measuring the duration of disfluencies and comparing it with the overall passage duration [7]. Both methods are time-intensive [8].

Given the compelling demand for effective detection of type of disfluencies, there has been an evident shift toward embracing innovative technologies, particularly artificial intelligence (AI) [9]. AI has the potential to substantially improve stuttering detection due to its advanced capabilities in processing and analyzing speech patterns. AI can handle large amounts of data and objectively process information. By training on extensive data containing both fluent and stuttered speech samples, AI models can learn to recognize distinct patterns associated with stuttering, such as repetitions, prolongations, and blocks.

This study aims to facilitate the identification and recognition of stuttering using deep learning (DL) models such as convolutional neural networks (CNNs) and convolutional long short-term memory (ConvLSTM). The proposed system can improve the precision and effectiveness of stuttering detection, supplementing conventional assessment approaches. By providing feedback and monitoring changes in speech patterns, the proposed system can improve the effectiveness and reliability of stuttering detection and evaluation.

The proposed work introduces a novel approach to enhance the accuracy of stuttering detection using the SEP-28K dataset along with a fluency bank dataset. This is achieved by employing upsampling methods to address limitations in the dataset and compensate for missing recordings. Unlike prior studies, our research uniquely integrates MFCC feature extraction with CNN and ConvLSTM models, a combination not previously explored. This innovative strategy provides fresh insights into automatic stuttering detection and holds promise for advancing the field.

The remainder of this paper is structured as follows: Section 2 reviews related work. Section 3 illustrates the proposed system. Section 4 presents the results and discussion. Finally, Section 5 concludes the study.

## 2. Related Work

Stuttering detection and classification have garnered considerable research interest, leading to a growing body of literature in the field. Numerous studies have been published that explored various methodologies and technologies to improve the identification and analysis of stuttering and disfluencies. Given the breadth of research contributions, reviewing these studies is essential to understand the current landscape, identify effective approaches, and highlight gaps that future work can address. Table 1 presents a comparative analysis of studies focused on stuttering, highlighting the dataset used, number of recordings, types of stuttering, feature extraction methods, classifiers employed, metrics analyzed, and results obtained.

Kourkounakis et al. [10] proposed an advancement in the detection and classification of stuttered speech by introducing a DL model that combined CNNs for feature extraction from spectrograms with bidirectional long short-term memory (BiLSTM) layers to capture temporal dependencies. Their approach outperformed existing methods, achieving a 26.97% lower miss rate on the UCLASS dataset than previous state-of-the-art methods and exceeding the performance of the unidirectional long short-term memory (LSTM) baseline for all stutter types. The authors focused on multiclass learning to address the complexity of stuttering, recognizing that multiple stuttering types can simultaneously occur within one utterance (e.g., “I went to uh to to uh to”). This perspective improves the robustness of the classification system, providing a more comprehensive understanding of stuttering variations and addressing a crucial gap in existing research on stuttered speech.

Sheikh et al. [11] proposed the StutterNet architecture, which is based on a time-delay neural network (TDNN) specifically designed to detect and classify various stuttering types. This architecture treats stuttering detection as a multiclass classification problem, incorporating components such as an input layer, time-delay layers, statistical pooling, fully connected layers, and a softmax layer. Their experimental results demonstrate that the StutterNet model achieves promising recognition performance across different stuttering types, even surpassing the ResNet + BiLSTM method in some instances, particularly in detecting fluent and non-fluent speech. StutterNet outperforms a state-of-the-art method based on a residual neural network and BiLSTM, achieving a notable gain of 4.69% in overall average accuracy and 3% in the Matthews correlation coefficient. The authors also outlined future work focusing on exploring the domain of multiple disfluencies, intending to investigate advanced variations in TDNNs for stuttering detection in real-world settings.

Kourkounakis et al. [12] introduced FluentNet in this regard, a deep neural network architecture specifically designed for automated stuttering speech detection. FluentNet includes components such as squeeze-and-excitation residual network (SE-ResNet) blocks, BiLSTM networks, and an attention mechanism, allowing it to achieve advanced results in stuttering detection and classification across various stuttering types on both the UCLASS and LibriStutter datasets. The model achieved an average miss rate of 9.35% and accuracy of 91.75% on the UCLASS dataset. In addition, the authors proposed experimenting with the architecture of FluentNet by potentially incorporating different attention mechanisms, including transformers, to further investigate their impact on the results.

Jouaiti and Dautenhahn [13] proposed a deep neural network that incorporates BiLSTM layers to detect stuttering and classify four types of disfluency. Their network architecture includes multiple components, such as dense layers, batch normalization, dropout layers, and an embedding layer for processing phoneme estimation data. The evaluation of their network on the FluencyBank dataset demonstrated its effectiveness and suitability for real-time applications, highlighting its potential as a valuable tool in speech therapy.

Al-Banna et al. [7] introduced a detection model that uses a two-dimensional (2D) atrous convolutional network specifically designed to learn spectral and temporal features from log mel spectrogram data. The architecture includes multiple layers, such as convolutional layers with varying dilation rates, batch normalization, dropout layers, and softmax activation, for predicting stuttering classes. Compared to existing stuttering detection methods, their model demonstrated superior performance, particularly in detecting prolongations and fluent speech. Their model outperformed state-of-the-art models in detecting prolongations, achieving F1 scores of 52% and 44% on the UCLASS and FluencyBank datasets, respectively. In addition, their model realized improvements of 5% and 3% in classifying fluent speech on both datasets, respectively. Al-Banna et al. also suggested the incorporation of atrous spatial pyramid pooling and local and global attention mechanisms to further increase detection scores.

Prabhu and Seliya [14] introduced a CNN-based automated stuttering identification system (ASIS) that distinguishes itself from previous models that rely on LSTM structures. Their CNN-based classifier demonstrated high accuracy and precision, achieving impressive F1 scores and surpassing existing models on various datasets. Notably, their model achieved the highest performance for interjections, obtaining an F1 score of 97.8%. The authors suggest that incorporating LSTM layers to capture temporal relations in data can further improve performance. In addition, they considered the possibility of utilizing different machine learning models, such as LSTM, for improved data interpretation on the SEP-28k dataset. Although the SEP-28k dataset is recognized for its robustness and substantial volume of stuttering data, which provides a valuable resource for model development, Prabhu and Seliya [14] highlighted opportunities for refinement and optimization. Their study underscores the potential of ASISs to substantially improve the quality of life of PWS, particularly in developing countries where SLPs are scarce.

Filipowicz and Kostek [15] conducted a comprehensive study on the automatic detection of stuttering and its subclasses, introducing a pitch-determining feature into their signal-processing toolkit while exploring various 2D speech representations and their influence on the classification results. Their evaluation included multiple classifiers, such as k-nearest neighbors (k-NN), support vector machines, and deep neural networks such as ResNet18 and Wav2Vec2. Notably, ResNet18 demonstrated superior performance, achieving an F1 measure of 93% for classifying speech disorders, which highlights the efficacy of DL approaches over classical methods. The authors proposed extending the training duration of each model and enhancing the ResNet18 architecture with additional convolutional layers, contingent on the available resources. However, they noted challenges in recognizing repeated sounds and words using the SEP-28K dataset, which was likely due to the limited number of positive examples for these categories.

A number of recent independent transformer-based detections have been proposed, such as Wav2Vec2, used in [16] for voice activity detection. Other transformer models, like HuBERT, have been applied to speaker emotion recognition, as demonstrated in [17], where it was used to identify emotions from speech. A new transformer model called TranStutter was proposed by Basak et al. [18] to classify various types of stuttering in audio recordings. Furthermore, Moghimi et al. [19] utilized GANBERT to detect stuttered words and categorize different stuttering types.

As shown in Table 1, the studies vary significantly in terms of dataset size, with the largest being SEP-28k, which includes 28,177 recordings, and the smallest being UCLASS, with 457 recordings. Different types of stuttering are addressed across the studies, such as sound and word repetitions or prolongations, utilizing feature extraction techniques like spectrograms and MFCCs. Classifiers range from deep residual networks combined with BiLSTM to CNNs and TDNNs, reflecting a variety of approaches in tackling stuttering classification.

In terms of performance metrics, the results show a wide disparity, with accuracy ranging from as low as 0.50 in [11] to as high as 0.95 in [14]. Notably, most studies have leaned towards applying deep learning models as their classification method. Additionally, the study using the SEP-28k dataset achieved the highest accuracy (0.95) and impressive precision (0.98), while the study with the lowest accuracy (0.50) also recorded the lowest precision and recall values. This comparison underscores the importance of dataset size and classifier choice in achieving reliable results in stuttering detection and classification.

## 3. Proposed System

This section outlines the process of developing an automated stuttering detection system, beginning with the selection and preparation of datasets for training. This section details the preprocessing steps required to prepare data for stuttering detection, followed by a discussion on the extraction of mel-frequency cepstral coefficient (MFCC) features from audio recordings. Next, the section discusses the construction of two classifiers and concludes with an evaluation of model performance.

### 3.1. Dataset

Various datasets have been employed in the field of automatic stuttering detection (ASD), including UCLASS, SEP-28k, FluencyBank, LibriStutter, and VoxCeleb, as mentioned in the literature review. Among these, the UCLASS and SEP-28k datasets have emerged as the most widely used in the literature. Notably, the SEP-28k dataset is one of the most recent publicly available datasets; therefore, we incorporated it into our study. In addition, in contrast to other publicly accessible stuttering datasets, SEP-28k stands out owing to its larger data size and inclusion of annotated information.

Considerable annotation effort involving at least three professionals improves the credibility of the labels and contributes to the robustness of models trained on this dataset [14]. In addition, we combine the FluencyBank and SEP-28k datasets because both datasets employ the same labeling system. Merging these datasets with consistent labels allows us to construct a larger and more diverse training dataset.

#### 3.1.1. SEP-28K Dataset

The SEP-28K dataset, introduced by Apple in 2021, contains 28,177 annotated audio samples from the Stuttered Events Podcasts series, marking the first publicly available dataset with stuttering-specific labels. These annotations classify speech disfluencies such as prolongations, repetitions, blocks, interjections, and fluent speech, as well as non-disfluent events like pauses, unintelligible segments, and poor audio quality [20]. Derived from 385 interviews across eight podcast series focused on individuals who stutter, the dataset is organized into approximately 3 s clips and managed through Python 3.13.0 scripts on GitHub [21]. Table 2 illustrates the types of stuttering and their description. As no Arabic stuttering datasets currently exist, this English dataset serves as a foundation, with future plans to develop a dedicated Arabic resource for stuttering research.

#### 3.1.2. FluencyBank Dataset

The FluencyBank AudioVisual dataset, which was created by Nan Bernstein Ratner from the University of Maryland and Brian MacWhinney from Carnegie Mellon University, was constructed to analyze the progression of speech fluency [22]. In our analysis, we employ annotations comparable to those used in the SEP-28K dataset, which are aligned with standards similar to Apple’s annotations for consistency and accuracy. Each clip received annotations for specific labels: “prolongation”, “block”, “sound repetition”, “word repetition”, and ”interjection”. These annotations were provided by three trained reviewers who, although not clinicians, were educated on how to identify each type of stuttering event. The label files include counts indicating how many of the three reviewers assigned each label to a clip (0, 1, 2, or 3). Multiple labels can be assigned to a clip [21].

### 3.2. Data Preprocessing

Initially, SEP-28K was intended to have 28,177 recordings; however, due to missing files, it currently contains only 21,855 recordings. To improve model accuracy, we merge the FluencyBank dataset with SEP-28K to handle the missing elements in SEP-28K. Jouaiti et al. [13] merged these two datasets, and their model achieved high results. By expanding our dataset through this integration, we obtained 32,000 recordings, thereby anticipating an improvement in model accuracy. Additional data provide more examples from which the model can learn, which generally improves performance, particularly in complex tasks where data variety is crucial for models to understand data nuances [23]. Moreover, training our CNN model separately on each dataset did not produce satisfactory results, as presented in Table 3.

#### 3.2.1. Merging Annotation

Herein, we combine annotation methods from the FluencyBank and SEP-28K datasets. We use annotation standards similar to those in the SEP-28K dataset, which includes 28,000 3 s clips, and align them with Apple’s protocols to ensure that the results are consistent and accurate. The FluencyBank dataset comprises 4144 3 s clips from 32 adults who stutter, with annotations that adhere to the same guidelines as those used in the SEP-28K dataset [20]. In total, the merged dataset contains 32,000 audio clips. Table 4 presents a sample of the records and annotations for the merged dataset prior to preprocessing. The different annotations, which are denoted as 0, 1, 2, or 3, represent the counts of how many of the three reviewers assigned each label to a particular audio clip.

#### 3.2.2. Annotation Balancing

**Figure 1:**
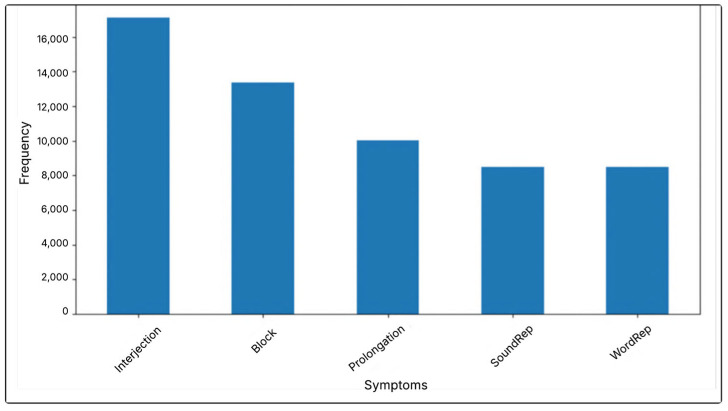
Distribution of label annotations in merged dataset.

The SEP-28k and FluencyBank datasets, similar to other speech datasets, suffer from an imbalance where certain classes are underrepresented [24]. Figure 1 illustrates the distribution of classes in the merged dataset, highlighting the prevalence of each category and offering insight into the imbalance between the different dataset classes.

As shown in Figure 1, the count of each label is as follows: Interjection is identified 17,128 times, Block is identified 13,381 times, Prolongation is identified 10,027 times, Sound Repetition appears 8494 times, and Word Repetition is identified 8486 times. This indicates class imbalance, which results in poor results, as reported in previous studies. In addition, the proposed model did not yield satisfactory results when applied to the dataset without appropriate upsampling.

**Figure 2:**
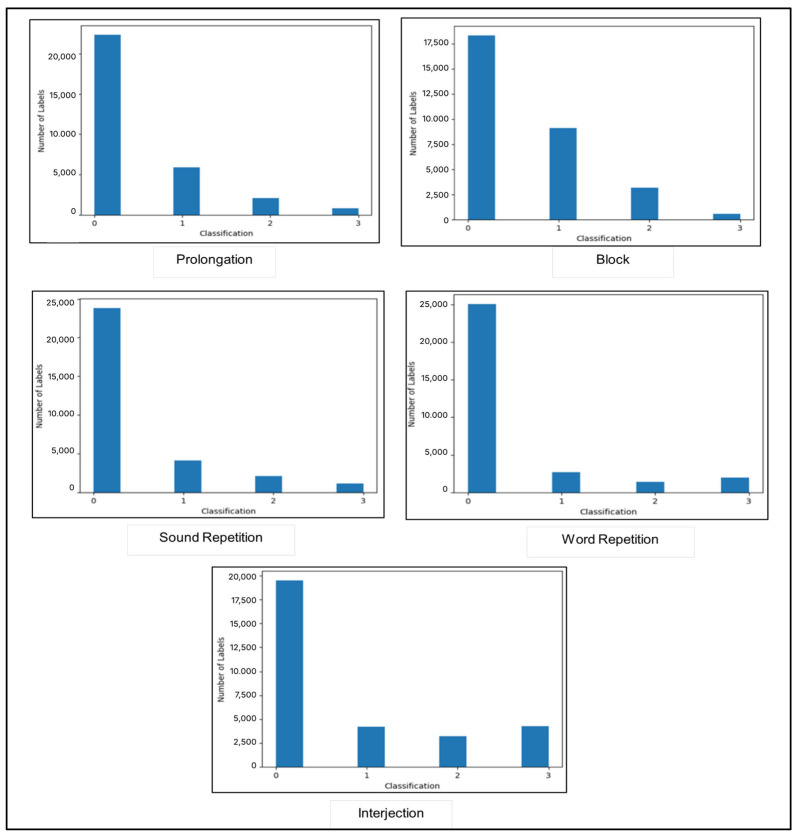
Distribution of each label annotation in merged dataset.

In addition, the dataset exhibits an uneven distribution, with values ranging from 0 to 3 representing the number of reviewers who identified each label. The frequency of zeros is higher than that of other values, indicating that certain labels, such as “block”, were rarely identified. Figure 2 illustrates the distribution of each label annotation in the merged dataset. This imbalance can negatively affect model performance. Similarly, the model developed by Filipowicz and Kostek [15] using the SEP-28K dataset encountered difficulty recognizing repeated sounds and words. This difficulty may be related to the small number of positive examples of these types in the dataset.

#### 3.2.3. Upsampling

We employed upsampling on the training dataset to balance the involved dataset, which substantially improved the learning capability of the proposed model. The experiments in [25] demonstrate that increasing the frequency of underrepresented sound events in the training data leads to considerable enhancements in accurately detecting and classifying those sound occurrences. In addition, the approach proposed by Tran et al. demonstrated the effectiveness of upsampling in improving predictive performance in healthcare applications, even with small datasets [26]. This technique helps ensure that models have sufficient examples of each class to learn from, which is important when some classes are underrepresented in the original data.

First, we segmented the dataset into smaller groups based on the combination of stuttering symptoms in each sample, such as prolongations, blocks, sound repetitions, word repetitions, and interjections. We created subsets for every possible combination of these symptoms, where “1” indicates that a symptom is present and “0” indicates that a symptom is absent. Then, we used the resample function to upsample each subset to obtain 1000 samples, thereby ensuring that each possible combination of symptoms had an equal representation in the balanced dataset. After all subsets were upsampled, they were combined into one balanced dataset and then shuffled to randomize the order of the samples. Table 5 presents a sample of the merged records and annotations after preprocessing.

**Figure 3:**
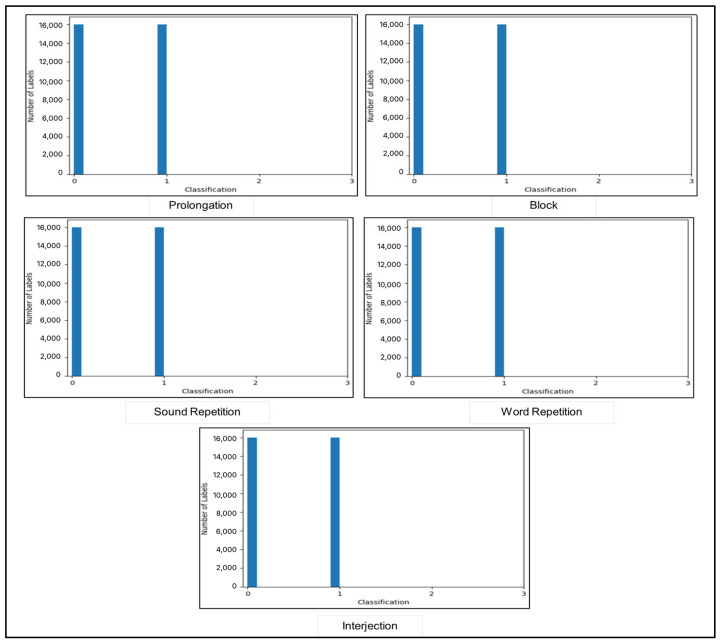
Distribution of label annotations in dataset after upsampling.

Figure 3 illustrates the distribution of label annotations in the training dataset after upsampling. The balanced dataset is composed of the last 13 columns containing MFCC features, while the remaining 5 columns represent the labels: “Prolongation”, “Block”, “Sound Repetition”, “Word Repetition”, and “Interjection”. Input features are then scaled using StandardScaler to achieve a mean of 0 and a standard deviation of 1. This scaling ensures that each feature contributes proportionally to the learning process, preventing any single feature from dominating due to differences in scale.

### 3.3. Feature Extraction

**Figure 4:**
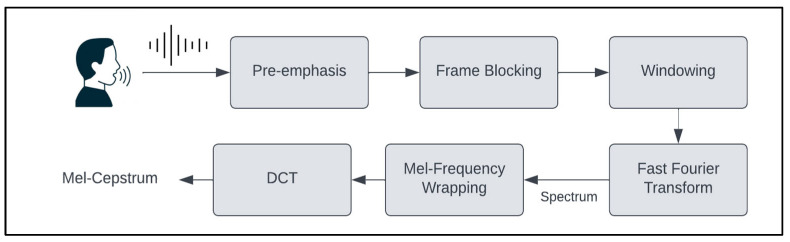
Steps of MFCC feature extraction.

In speech processing, feature extraction involves capturing critical information from a speech signal while effectively reducing noise and irrelevant components [27]. This process is crucial for differentiating between various speech patterns [28]. The MFCC method is the primary and highly effective technique for speech-related applications [29]. It achieves this by incorporating a logarithmic function and mel filters to replicate the human auditory system and using triangular bandpass filters to convert frequency information, thereby simulating the way humans perceive sound [30]. The steps of MFCC feature extraction include pre-emphasis, frame blocking, windowing, fast Fourier transform (FFT), mel-frequency wrapping, and discrete cosine transform (DCT). The steps are illustrated in Figure 4.

In this study, we extracted 13 cepstral coefficients from each signal using the librosa library to compute the MFCCs. The cepstrum, also known as the cepstral coefficient, is commonly employed as the acoustic feature and is defined as the inverse Fourier transform of the short-time logarithmic amplitude spectrum [31]. In general, the initial 13 coefficients of the MFCCs are used as features because they represent the spectral envelope. The higher dimensions that are omitted contain finer details of the spectral information. Selecting many cepstral coefficients increases model complexity [32].

Step 1: Pre-emphasis: The initial step involves passing the signal through a pre-emphasis filter to enhance the energy at higher frequencies. This pre-emphasis filter is beneficial because it harmonizes the frequency spectrum, addressing the typical discrepancy where high frequencies tend to have lower energy magnitudes than lower frequencies [32].

Step 2: Frame Blocking: The speech signal is divided into brief time intervals, typically spanning 20–30 s, which are referred to as frames. The voice signal is partitioned into N samples, and adjacent frames are separated by a gap denoted as M, where M is typically set to 100 and N to 256. This framing process is essential because speech is a signal that evolves; however, when it is observed over short intervals, its characteristics remain relatively constant. Consequently, short-time spectral analysis is performed [33].

Step 3: Windowing: Windowing minimizes spectral distortion by gradually reducing the signal amplitude to zero at the start and end of each frame. The Kaisar_fast windowing was utilized. The resulting signal is acquired by multiplying the original signal by a window function at time n [34].

Step 4: Fast Fourier Transform: FFT is used to transform information from the time domain to the frequency domain. To acquire the magnitude frequency representation for each frame, we use FFT. The FFT outcome is a spectrum or periodogram [33].

Step 5: Mel-Frequency Wrapping: In this step, the calculated spectra are mapped to the mel scale using triangular filters. These filters are adjusted in terms of spacing and width based on the mel-frequency scale, which accommodates variations in human perception of frequency. Below 1000 Hz, the mapping remains linear; however, it becomes logarithmic above 1000 Hz [35].

Step 6: Discrete Cosine Transform: DCT is employed to extract time-domain information from log Mel spectra. The resulting DCT output yields MFCCs [29].

### 3.4. Classification Model

In this study, we employed two DL models: CNN and ConvLSTM. The primary objective is to apply these models to the automated identification and categorization of various types of stuttering. CNNs are particularly effective for analyzing spatial hierarchies within data, rendering them highly suitable for tasks that involve feature extraction from audio signals. In contrast, ConvLSTM networks integrate convolutional layers with LSTM units, enabling the model to capture both spatial and temporal dependencies in sequential data. This capability is essential for the accurate detection and classification of stuttering events [36]. In the following, we explain the experiments conducted to automatically detect stuttering types in Section 3.4.1 and Section 3.4.2.

#### 3.4.1. Convolutional Neural Networks

CNNs stand out as a groundbreaking DL approach, successfully robustly training numerous layers. This architecture, known for leveraging spatial relations, reduces the number of parameters to learn, thereby enhancing the efficiency of general feedforward backpropagation training. CNNs were introduced with a focus on minimal data preprocessing requirements, which contributes to their versatility and effectiveness [37].

In this study, the proposed model was used to identify the type of stuttering among “Prolongation”, “Block”, “Sound Repetition”, “Word Repetition”, or “Interjection”. The CNN model outputs the probabilities for each label using separate sigmoid activation functions in the output layers. This configuration is typical for multilabel classification tasks, where each label/class is considered independently, and the sigmoid function is used to predict the probability that a particular class/label is present [38].

The implementation of CNNs involves three main types of layers: the input layer of extracted features, hidden layers, and the output layer for multilabel classification.

**Figure 5:**
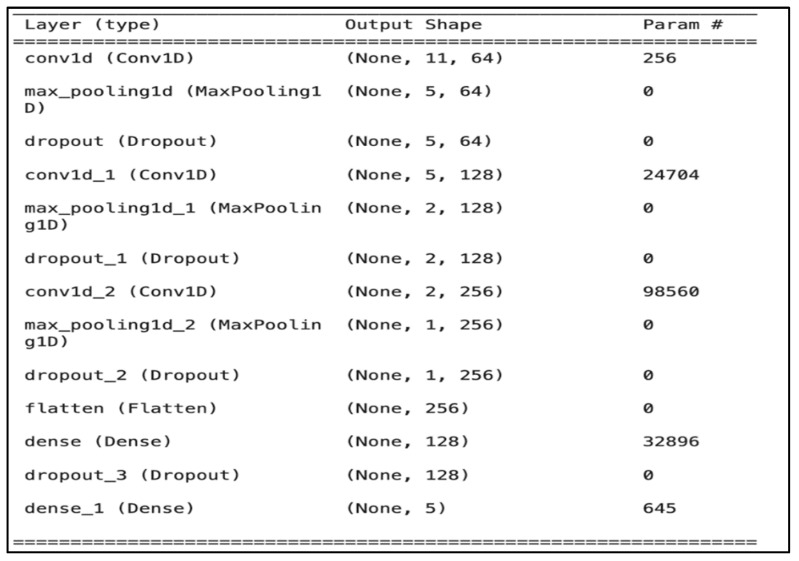
CNN model structure.

- Input layer: This layer serves as input features for the CNN model, which comprises 13 columns, which are the MFCC features.
- Hidden layers: The proposed model comprises multiple layers (Figure 5), including (i) three convolutional, (ii) four dropout, (iii) three max pooling, (iv) one flatten, and (v) one fully connected (dense) layer.

The first convolutional layer of our CNN model inputs 13 MFCC features formatted as (13, 1), treating each feature as a separate sequence element. This layer uses a kernel size of 3 and strides of 1 to analyze three features at a time, summarizing them into new features [39]. “Valid padding” is applied, causing the kernel to stop sliding when it can no longer fit fully, reducing the output from 13 to 11 features [40]. Subsequent layers use “same padding”, adding zeros to maintain the dimensionality unless changed by pooling. Thus, the output of the first layer consists of 64 new feature sets, each reduced to 11, enabling the model to recognize diverse audio patterns.

The first layer applies 64 filters, the second applies 128 filters, and the last applies 256 filters. A pooling layer reduces the output size, followed by two additional pooling layers to further decrease parameters. To prevent overfitting, three dropout layers are implemented before the dense layer, with one more after it. A flattening layer converts the output from the convolutional layers into a one-dimensional array, and a fully connected layer with 128 units processes the data, using ReLU activations in the initial layers and sigmoid activation in the final layer to produce a probability distribution.

- Output layer: Separate dense layers with sigmoid activation functions are used for each label for multilabel classification of five labels, “Prolongation”, “Block”, “Sound Repetition”, “Word Repetition”, and “Interjection”. Then, we adjust the learning rate to improve model training. For training, separate models were trained for each label with binary cross-entropy loss, and their accuracy was evaluated. The model predictions are aggregated using a moving average formula, resulting in a probability vector representing the likelihood of each class. The class with the highest score is the predicted class. Inference times for each stuttering type trained on the CNN model are shown in Table 6.

#### 3.4.2. Convolutional Long Short-Term Memory

Recently, BiLSTM has achieved superior recognition rates in acoustic modeling, which can be attributed to its ability to reinforce higher-level representations of acoustic data. The recognition rate is notably influenced by the crucial spatial and temporal properties of speech signals. Therefore, the integration of LSTM and CNNs can realize superior performance [36]. ConvLSTM incorporates a sequence of convolutional layers prior to the recursive layer, which is tasked with recognizing complex patterns within input data [15]. During the convolution operation, ConvLSTM captures spatiotemporal cues, which improves its ability to extract correlations among distinct speech segments [41].

The implementation of our ConvLSTM model involves three main types of layers: the input layer of extracted features, hidden layers, and the output layer for multilabel classification.

**Figure 6:**
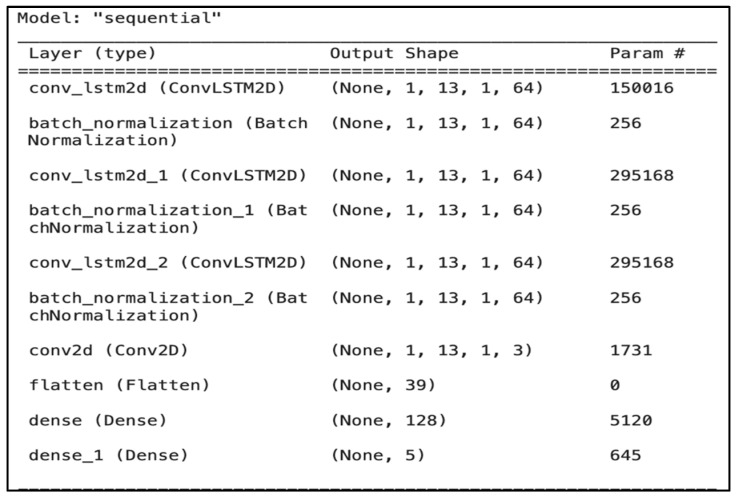
ConvLSTM model structure.

- Input layer: Similar to the previously described CNN input layer, the ConvLSTM also takes 13 MFCC features.
- Hidden layers: The model architecture (Figure 6) comprises (i) a pair of ConvLSTM2D layers, (ii) three batch normalization layers, (iii) a single convolutional layer, (iv) a flatten layer to reshape the data, and (v) two dense layers for output processing. Using ConvLSTM2D layers, the model captures the temporal dynamics and spatial characteristics of sequential audio data. The batch normalization layers follow each ConvLSTM2D layer to normalize the activations, which accelerates learning and leads to faster convergence.
- Output layer: The model comprises a Conv2D layer designed to handle the 2D data structure output by the ConvLSTM2D layers. The flatten layer then converts the 2D data structure into a 1D array suitable for input into the dense layers. The first dense layer with ReLU activation serves as a fully connected layer that learns nonlinear combinations of features. The final dense layer uses a sigmoid activation function to output probabilities for each class, essentially performing multilabel classification. The average inference time per sample in batch mode was 0.97 ms, while the average inference time for a single sample was 120.31 ms.

### 3.5. Model Evaluation

For ConvLSTM and CNN, we split the audio signals into 70% training, 20% validation, and 10% testing sets. For other classifiers, the data were partitioned into 70% training and 30% split between validation (67%) and testing (33%). To understand the results of our models and demonstrate that they do not have an overfitting problem, we plotted the training and validation losses. Table 7 (left panel) shows the training and validation losses for our CNN model during training for 100 epochs.

The blue and orange lines represent the training and validation losses, respectively. Initially, both losses decrease rapidly, which suggests that the model learns quickly from the training data. As the number of epochs increased, the rate of loss reduction decreased for both lines, and they began to plateau. This indicates that the model was starting to converge, which implies that with each epoch, the model slightly improves on the training data.

The training loss appears to be consistently lower than the validation loss, which is expected because the model is directly learning from the training data. However, the proximity of the two lines suggests that the model is generalizing well; the validation loss is not considerably higher than the training loss, which can be a sign that models will perform well on unseen data. At the end of 100 epochs, both lines flatten out, which indicates that further training yields minimal improvements. It is also worth noting that the validation loss follows a pattern similar to that of the training loss throughout the training process, which indicates that the model does not overfit the training data. The graph shows a desirable training outcome, with both losses decreasing and stabilizing.

In addition, the training and validation loss curves of our ConvLSTM model are presented in Table 7 (right panel). During the training phase, a rapid decrease in the training loss is observed (the blue line), suggesting that the model is learning effectively from the dataset. The validation loss, which is indicated by the orange line, decreases initially but begins to exhibit a consistent and uneven trend as the number of epochs increases.

The stabilization of the validation loss indicates a point at which the performance improvement of the model on the validation data degrades. The fluctuations demonstrate that the learning of the model is less stable on data on which it is not trained. Although this behavior is not necessarily a concern, it highlights the complexity of the learning dynamics of the model as it processes and learns from the data over time.

The performance of the CNN model across the labels is illustrated in confusion matrices in Table 8, indicating a strong ability to classify both the presence and absence of the type of stuttering. For the Prolongation label, the CNN model correctly identified 1496 true negatives and 1537 true positives with few errors. The Block label exhibited a similar pattern, with 1501 true negatives and 1537 true positives, although there were slightly more misses. The SoundRep label exhibited an impressive detection rate with 1554 correct non-occurrences, 1539 occurrences, and minimal misclassifications.

WordRep stands out with the highest number of true positives (1601) and only four misses, demonstrating remarkable accuracy. Finally, the Interjection label also reported notable true positives and negatives, 1572 and 1510, respectively, demonstrating the consistent classification ability of the model across different contexts. Overall, the low numbers of false positives and false negatives across all labels reflect the proficient generalization and discernment capabilities of the model.

Table 8 presents the confusion matrices for our ConvLSTM model across all labels. The confusion matrices show several true positives and true negatives across all labels, which highlight the strong classification abilities of the ConvLSTM model. In particular, for Prolongation, there were 1493 true negatives and 1541 true positives, and for Block, there were 1481 true negatives and 1531 true positives.

The model performed similarly well for SoundRep (1530 true negatives and 1545 true positives) and even better for WordRep (1522 true negatives and 1602 true positives). For Interjection, the ConvLSTM model identified 1499 true negatives and 1563 true positives. False negatives and positives were kept to a minimum across all labels, with the highest number being 72 false positives for Interjection and the lowest being 3 false negatives for WordRep, demonstrating the precise predictive performance of the model and its ability to generalize well from the training data.

## 4. Results and Discussion

In addition to the confusion matrix results, we compared our CNN and ConvLSTM models to the model proposed by Prabhu and Seliya [14]. In addition, we implemented several classical models, including K-nearest neighbors (KNN), decision tree, and random forest. By analyzing and contrasting the accuracy, precision, recall, and F1 scores across our models, we obtained a detailed understanding of their efficacy and determined the most effective method for producing precise predictions. The results of our implementation showed significant improvement following the application of several processing steps, including the merging of two datasets and the use of upsampling methods, which notably enhanced accuracy. dditionally, our decision to utilize MFCC extraction proved highly effective, as it emerged as one of the best-performing feature extraction techniques for our methodology. Table 9 presents the performance metrics for comparing the two models, along with the benchmark model [14], including accuracy, precision, recall, and F1 score and their 95% confidence intervals.

According to the results, our CNN model, when applied to the Word Repetition, outperforms the others across all metrics. It achieves the highest accuracy, precision, recall, and F1 score, demonstrating its superior ability to correctly identify instances of word repetition stuttering. For the Prolongation and Block labels, the models exhibit high and comparable performances. However, our ConvLSTM model exhibits a slightly lower recall for Block. This suggests that the model is less adept at capturing all actual instances of Block stuttering than our CNN model. For Sound Repetition, the models generally perform well. The benchmark model [14] demonstrates exceptional precision and recall for the Sound Repetition label. This indicates a high level of accuracy and comprehensive identification of Sound Repetition stutter events using this model.

However, when evaluating the Word Repetition label, the benchmark model exhibits a noticeable decrease in precision and recall, suggesting a potential weakness or compromise in its ability to classify Word Repetition stuttering compared to its performance on Sound Repetition. In contrast, our models demonstrate superior capabilities for this label. Specifically, the CNN model exhibits remarkably higher precision and recall values for Word Repetition, indicating stronger proficiency in accurately classifying this type of stuttering without as many false positives or negatives. This contrast is particularly evident when comparing the near-perfect precision of our CNN model (0.997) to the lower precision of the benchmark model for Word Repetition. Similarly, the recall of our CNN model is higher, which suggests that it is more effective at identifying most actual Word Repetition instances. Overall, this result highlights the more balanced and effective approach of our CNN model for handling Word Repetition stuttering compared to the benchmark model, which appears to struggle in this stuttering category. For Interjection, both the CNN model and the benchmark model exhibit high precision and recall.

Comparing our ConvLSTM and CNN models across various stuttering types, the CNN model generally performs better. For Prolongation, both models achieve similar results; however, the CNN model slightly outperforms the ConvLSTM model in terms of precision. For Block, the CNN model scores slightly higher in accuracy and precision. The difference is more noticeable for Sound Repetition, where the CNN model exhibits considerably better precision even though its recall is slightly lower. For Word Repetition, the CNN model stands out notably in terms of precision and recall, which indicates that it is more accurate and misses fewer actual cases. For Interjection, the CNN model exhibits higher precision and overall F1 score despite a small compromise in recall.

To further evaluate the performance of our model, we implemented the following classical models: random forest, decision tree, and KNN with five neighbors. Table 10 presents the performance metrics for comparing these models, including accuracy, precision, recall, and F1 score, and their 95% confidence intervals.

Among these conventional models evaluated for stuttering classification, the random forest classifier demonstrated superior performance compared to the decision tree and KNN models. Specifically, the random forest model excelled at classifying Word Repetition with impressive scores of 95% across the accuracy, precision, recall, and F1 metrics, and it performed notably well in Sound Repetition, with scores of approximately 94% for most metrics. The random forest model exhibited consistently high performance across all types of stuttering, particularly standing out in handling more complex stuttering patterns such as Word Repetition, for which detection precision is crucial.

Conversely, the decision tree and KNN models exhibited lower performance, with the decision tree model scoring approximately 89–91% for various stuttering types, and the KNN model falling behind, with scores primarily in the 80% range. The lower performance of the KNN model can be attributed to its sensitivity to the data configuration and size, which can affect its ability to generalize effectively across different stuttering types. This analysis highlights random forest as the best choice among the three models for stuttering classification because of its robustness and higher accuracy in distinguishing between different stuttering phenomena.

## 5. Conclusions

Accurate detection of type of disfluencies in conversation speech is crucial for understanding the type of disfluencies in speech. This study proposed an automated detection system to identify different types of disfluencies quickly and accurately. If developed systems prove robust in detecting disfluencies, they can be used as assisting tools for the SLPs in assessment and management.

During the design and implementation of this study, we noted that available speech data exhibited notable imbalances, which complicated the development of robust models. To address this issue, we employed techniques such as upsampling and data integration, which improved model performance.

## Figures and Tables

**Table 1:** Summary of related studies and results.

Study	Dataset	Number of Recordings	Type of Stuttering	Feature Extraction	Classifier	Metric	Result
[10]	UCLASS	457	Sound repetition, word repetition, phrase repetition, revision, interjection, and prolongation	Spectrograms	Deep residual network (ResNet) + BiLSTM	Accuracy	0.91
						Miss rate	0.10
[11]		457	Average of block, fluent, repetition, and prolongation	MFCC	Time-delay neural network (TDNN)	Accuracy	0.50
						Precision	0.45
						Recall	0.38
						F1 score	0.39
[12]	UCLASS	457	Average of sound, word, and phrase repetitions, revisions, interjections, and prolongations	Spectrograms	SE-ResNet + attention mechanism + BiLSTM	Accuracy	0.917
	LibriStutter	4736				Miss rate	0.093
[13]	FluencyBank	4144	Average of word repetition, sound repetition, interjection. and prolongation	MFCC	BiLSTM	Accuracy	0.84
						Recall	0.70
						F1 score	0.70
[7]	UCLASS	457	Average of block, repetition, interjection, and prolongation	Mel spectrogram	CNN, atrous convolutional network	Recall	0.50
						F1 score	0.50
	FluencyBank	4144				Recall	0.51
						F1 score	0.56
[14]	SEP-28k	28,177	Average of blocks, prolongations, sound repetitions, word repetitions, and interjections	Spectrograms	CNN	Accuracy	0.95
						Precision	0.98
						Recall	0.88
						F1 score	0.93
[15]		28,177	Average of prolongation, block, sound repetition, word repetition, interjection, and disrupted speech	MFCC	ResNet18	F1 score	0.93

**Table 2:** Types of stuttering.

Type	Definition	Example
Prolongation	Extending or elongating sounds or syllables within words.	“Sssssend me that email, please”.
Block	Temporary interruption or cessation of speech flow.	“I can’t… go to the… park tonight”.
Interjection	Spontaneous and abrupt interruption in speech with short exclamations.	“Um, I don’t know the answer”.
Sound repetitions	Repeating individual sounds within a word.	“Th-th-that movie was great”.
Word repetitions	Repeating entire words within a sentence.	“I like pizza, pizza, pizza”.

**Table 3:** Training CNN model across SEP-28K and FluencyBank separately.

Classifier	Dataset	Type of Stuttering	Accuracy
CNN	Sep28K	Prolongation	69.1%
		Block	30.9%
		SoundRep	76.6%
		WordRep	80.2%
		Interjection	13.4%
	FluencyBank	Prolongation	70.8%
		Block	63.7%
		SoundRep	72.4%
		WordRep	65.9%
		Interjection	28.6%

**Table 4:** Samples of merged records and annotations before preprocessing.

Audio Clip	Prolongation	Block	SoundRep	WordRep	Interjection
FluencyBank_107_3	1	0	0	0	3
HeStutters_3_10	2	1	0	0	0
HVSA_3_76	3	0	0	0	0
MyStutteringLife_3_31	0	1	1	0	0

**Table 5:** Sample of merged dataset after preprocessing.

Audio Clip	Prolongation	Block	SoundRep	WordRep	Interjection
FluencyBank_107_3	1	0	0	0	1
HeStutters_3_10	1	1	0	0	0
HVSA_3_76	1	0	0	0	0
MyStutteringLife_3_31	0	1	1	0	0

**Table 6:** CNN inference times.

Stuttering Type	Batch Inference Time
Prolongation	0.36 s
Block	0.69 s
SoundRep	0.36 s
WordRep	0.69 s
Interjection	0.68 s

**Table 7:** Total loss for CNN and ConvLSTM models.

CNN	ConvLSTM
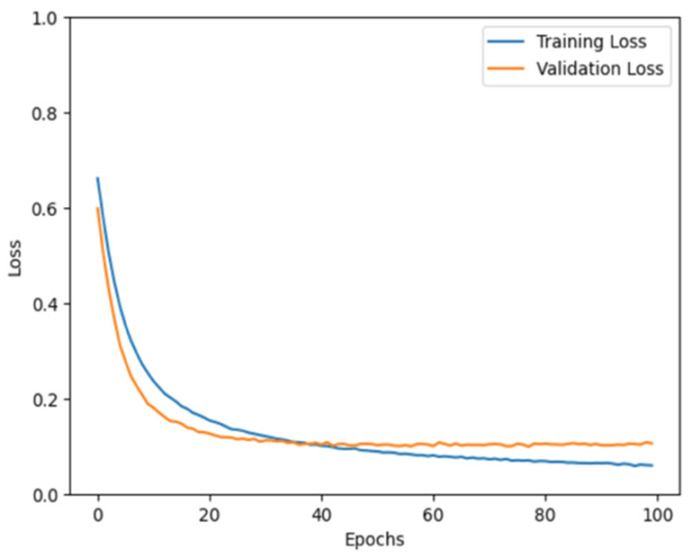	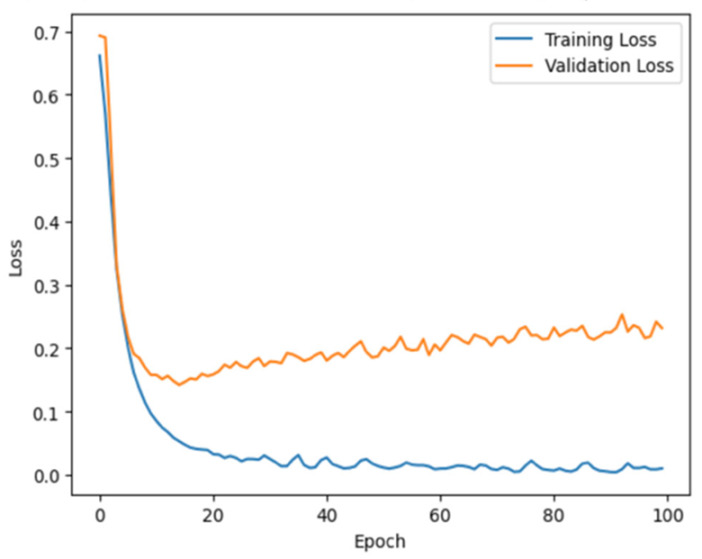

**Table 8:** Confusion matrices for CNN and ConvLSTM models.

Type	CNN	ConvLSTM
**Prolongation**	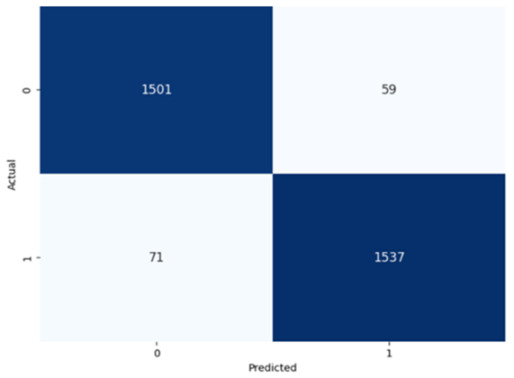	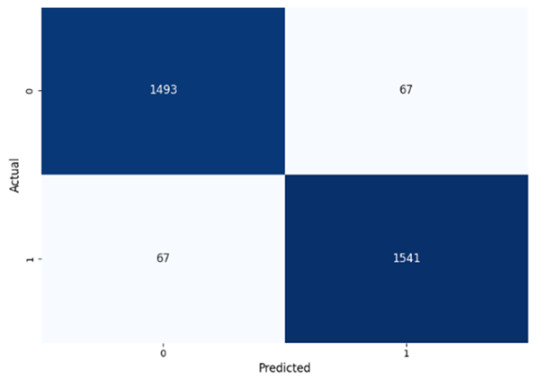
**Block**	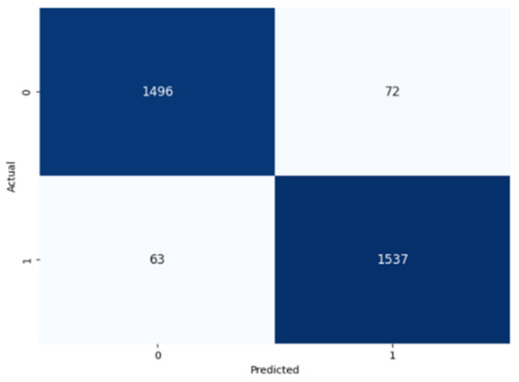	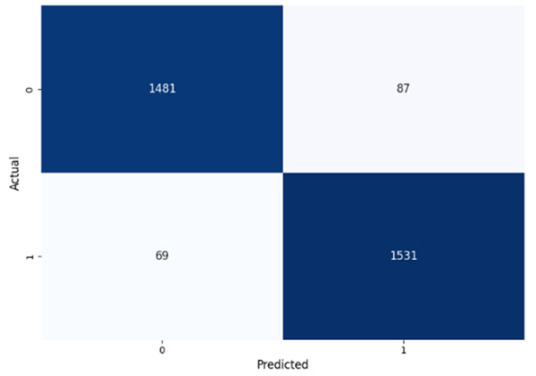
**Sound repetitions**	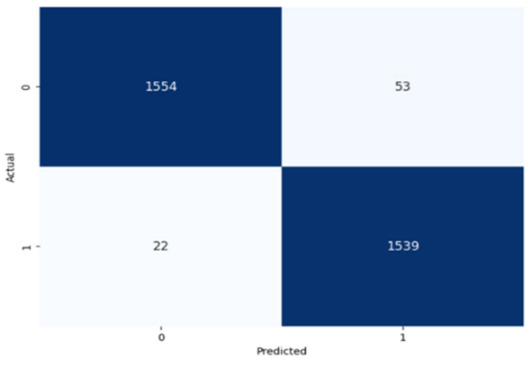	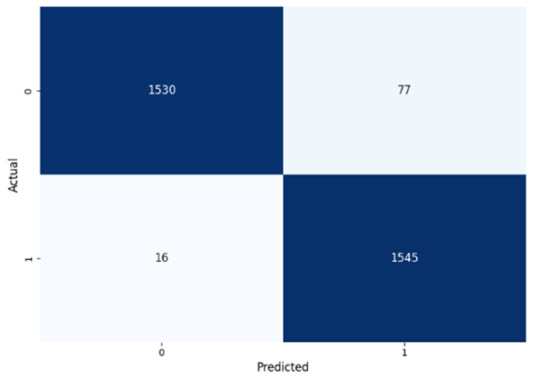
**Word repetitions**	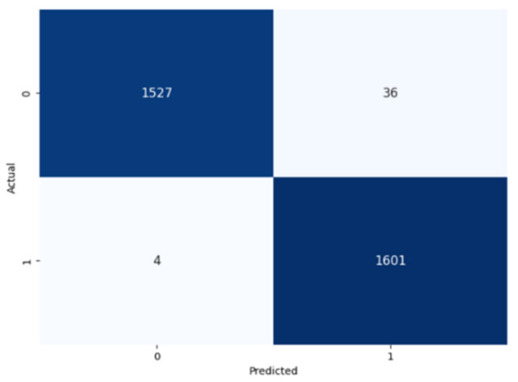	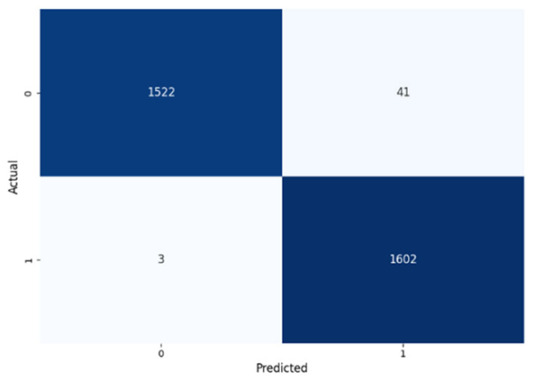
**Interjection**	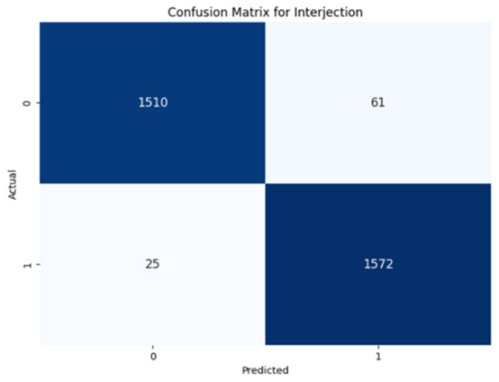	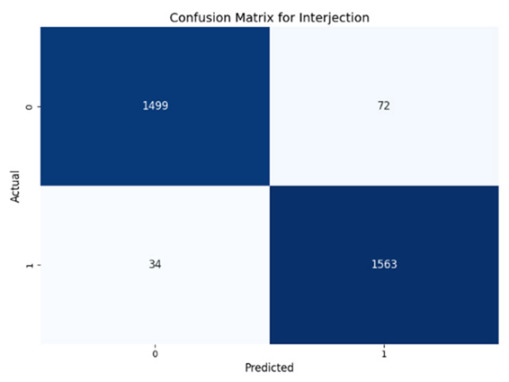

**Table 9:** Performance metrics on SEP-28K and FluencyBank datasets.

Feature Extraction, Classifier	Type of Stuttering	Accuracy	Precision	Recall	F1
MFCC + CNN	Prolongation	95.8%	95.4%	96.2%	95.8%
	Block	95.7%	95.9%	95.4%	95.6%
	SoundRep	97.6%	98.6%	96.7%	97.6%
	WordRep	98.7%	99.7%	97.6%	98.7%
	Interjection	97.2%	98.3%	96.1%	97.2%
MFCC + ConvLSTM	Prolongation	95.7%	95.8%	95.8%	95.8%
	Block	95.0%	94.6%	95.6%	95.1%
	SoundRep	97.0%	95.2%	98.9%	97.0%
	WordRep	98.6%	97.5%	99.8%	98.6%
	Interjection	96.6%	95.5%	97.8%	96.7%
Benchmark [14] Spectrograms + CNN	Prolongation	96.8%	98.4%	86.2%	91.8%
	Block	93.4%	99.8%	83.9%	91.2%
	SoundRep	96.8%	99.8%	98.9%	97.0%
	WordRep	98.6%	85.4%	86.7%	92.6%
	Interjection	96.6%	95.7%	99.9%	97.9%

**Table 10:** Performance metrics of traditional models.

Classifier	Type of Stuttering	Accuracy	Precision	Recall	F1
Decision Tree	Prolongation	89%	89%	89%	89%
	Block	89%	89%	89%	89%
	SoundRep	91%	91%	91%	91%
	WordRep	91%	91%	91%	91%
	Interjection	89%	89%	89%	89%
Random Forest	Prolongation	91%	92%	91%	91%
	Block	89%	89%	89%	89%
	SoundRep	94%	94%	94%	93%
	WordRep	95%	95%	95%	95%
	Interjection	91%	91%	91%	91%
K-Nearest Neighbors (KNN)	Prolongation	80%	80%	80%	80%
	Block	79%	79%	79%	79%
	SoundRep	80%	80%	80%	80%
	WordRep	82%	82%	82%	82%
	Interjection	80%	80%	80%	80%

## Data Availability

The code, along with the balanced dataset, is published on GitHub. https://github.com/Raghad-Alnashwan/StutteringDetection. accessed on 1 May 2025.
